# A primer on deep learning and convolutional neural networks for clinicians

**DOI:** 10.1186/s13244-021-01052-z

**Published:** 2021-08-12

**Authors:** Lara Lloret Iglesias, Pablo Sanz Bellón, Amaia Pérez del Barrio, Pablo Menéndez Fernández-Miranda, David Rodríguez González, José A. Vega, Andrés A. González Mandly, José A. Parra Blanco

**Affiliations:** 1 Advanced Computation and e-Science, Instituto de Fsica de Cantabria - CSIC, Santander, Spain; 2 Servicio de Radiodiagnostico, Hospital Universitario Marques de Valdecilla, Santander, Spain; 3 Instituto de Investigación Sanitaria Valdecilla (IDIVAL), Santander, Spain; 4 Departamento de Morfologa y Biologa Celular, Universidad de Oviedo, Oviedo, Spain; 5 Facultad de Ciencias de la Salud, Universidad Autonoma de Chile, Santiago de Chile, Chile

**Keywords:** Deep learning, Image processing, Medical imaging, Educational

## Abstract

Deep learning is nowadays at the forefront of artificial intelligence. More precisely, the use of convolutional neural networks has drastically improved the learning capabilities of computer vision applications, being able to directly consider raw data without any prior feature extraction. Advanced methods in the machine learning field, such as adaptive momentum algorithms or dropout regularization, have dramatically improved the convolutional neural networks predicting ability, outperforming that of conventional fully connected neural networks. This work summarizes, in an intended didactic way, the main aspects of these cutting-edge techniques from a medical imaging perspective.

## Introduction

Artificial intelligence (AI) is defined as the intelligence demonstrated by machines, in contrast to the natural intelligence displayed by humans. Despite the hype that is currently encountering, it is not something as new as one may imagine. Most of the people consider that the historical article written by Turing in 1950 [1] established the beginning of this new field by asking a simple question: *Can machines think?* AI includes what was later called Machine Learning, but the first AI algorithms did not actually learn. The first AI computer programs were based on the so-called symbolic AI. This first approach consisted on programming a set of rules large enough to manipulate knowledge and reached its height of popularity during the boom of expert systems in the eighties. Symbolic AI was probed to work fine for logic problems where the rules were clear, such as chess playing, but were useless for more diffuse and perceptual problems such as the recognition and manipulation of images, voice, language. This is where the *learning* approach comes into play.

## The learning approach

The concept of machine learning arose from the need of answering certain questions that were not covered by the symbolic AI, where all the rules to solve certain problem should be coded by some expert. Some of these open issues were:

- Can a computer program go beyond what we know how to code?
- Can a computer program learn just by looking at the data?
- Can a computer program even surprise us?

**Fig. 1:**
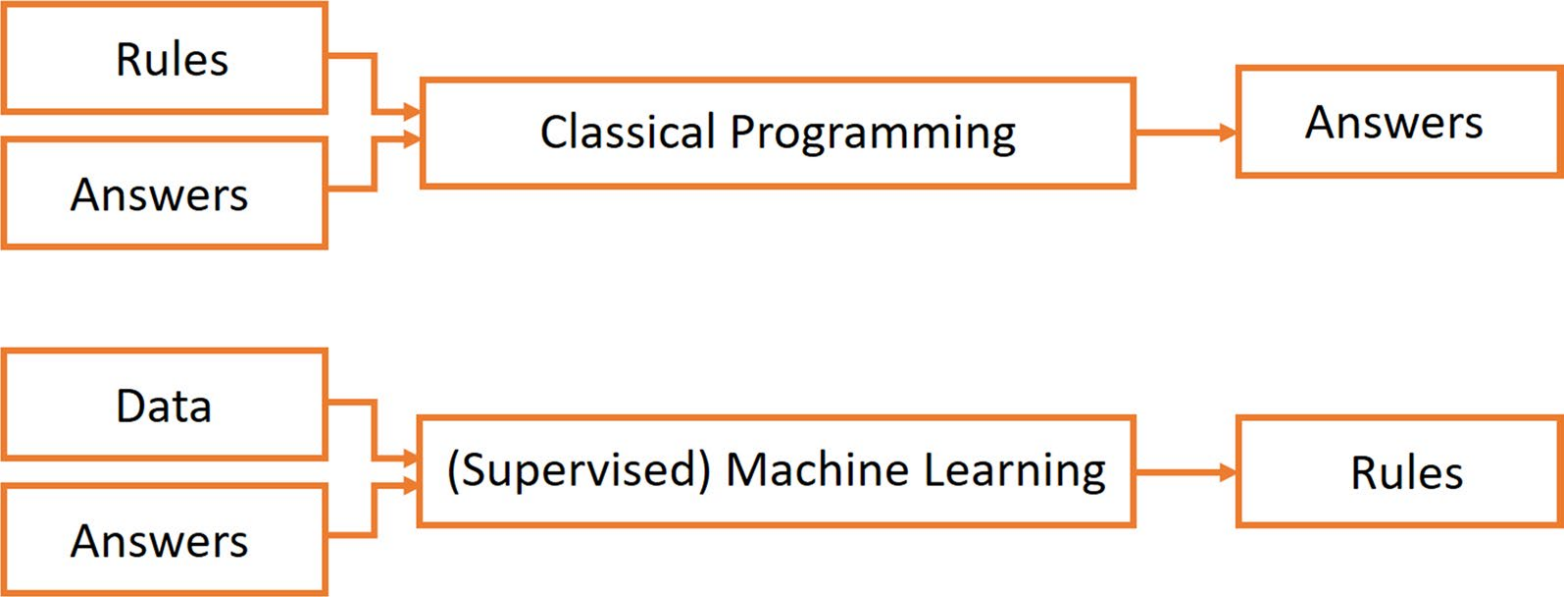
Classical programming vs supervised learning approach

The main idea behind Machine Learning is to let the computer learn directly by exposing it to a large number of examples of a given situation or class. The Machine Learning algorithm will then automatically develop a model that can deduce and generalize the examples it was exposed to and make predictions from it for totally new cases. This allows to develop systems capable of tasks so diverse as predicting house prices by *looking* at the historical behavior of the real-estate market (regression problem) or a system that is able to learn how to distinguish two different varieties of glioblastoma that it has never seen before just by *looking* at many different samples of magnetic resonance images (MRI) of both categories (classification problem). At the end of the day, this is similar to the way in which we humans learn: by exposition to many examples allowing us to generalize a certain concept. In the context of medical imaging, there are currently two main different types of learning: supervised learning and unsupervised learning. In the supervised learning approach, the learning algorithm receives as input a series of data tagged with the correct answer or label. This means that, in the case of the glioblastoma classification, the machine learning system would have access to several MRI of each of the two glioblastomas types with a label for each of them indicating to which category it belongs. One can easily see the improvement with respect to the symbolic AI: in the symbolic AI approach, some expert should have found and coded the rules allowing to distinguish the two different types of tumors. This often implies having a well-established metric on how to distinguish the categories to be predicted, something that is usually not the case in computer vision problems. Actually, most of the mechanisms used by humans to perform daily actions, such as recognizing faces or even speaking or understanding the context of a sentence, would be very difficult to wrap up into some coded rules. The paradigm shift between classical programming or symbolic AI and the machine learning approach can be easily understood by looking at Fig. 1.

In the unsupervised learning approach, the learning algorithm does not receive the labels. Instead, it only receives some input data and the algorithm alone will work on its own to extract the information needed to solve the problem under study. One of the most common tasks to be solved with an unsupervised approach is the clustering. It consists in grouping a set of objects in such a way that objects in the same group (called a cluster) are more similar, according to a certain metric, to each other than to those in other groups. For the glioblastoma case previously mentioned, this would imply to just give the algorithm as input a set of brain MRI without any further indication and let the system learn whether there are some meaningful features allowing to separate the dataset into categories. This can be an easy task if we want to separate very obvious categories such as images of green circles from images of red circles, but can become really complicated for more multidimensional tasks. Some of the advantages of the unsupervised learning approach is that it could allow us to really learn new things about the problem. For instance, it may find three different tumor categories instead of two, potentially allowing us to discover nuances in brain cancer diagnosis that escaped the human experience. Besides, it is much easier to find unlabelled data than finding some expert willing to tag a big dataset for training a supervised algorithm. On the other hand, the main disadvantage of the unsupervised learning is that, nowadays, the algorithms are still less accurate and trustworthy than the supervised methods.

It is important to note that, even if we have focused on supervised and unsupervised learning, there are other learning approaches less used for now in the medical context, but still worth mentioning:

- The semi-supervised learning approach combines both supervised and unsupervised learning. In this case, the algorithm can learn from a mixture of labelled and unlabelled data. This approach includes a range of possible techniques that are outside the scope of this paper.
- The reinforcement learning approach is concerned with how software agents should take actions in an environment by maximizing some portion of a certain cumulative reward. These are goal-oriented algorithms, which learn how to attain a complex objective (goal) or how to maximize along a particular dimension over many steps. For example, they can maximize the points won in a game over many moves. This approach is widely used in robotics nowadays.

In this section, the different types of learning available for machine learning algorithms have been summarized. For the examples in this article, we will be focused on classification problems using the supervised learning approach since it is the most widely used nowadays in medicine as it has proved its success for many different applications.

## How computers see images

**Fig. 2:**
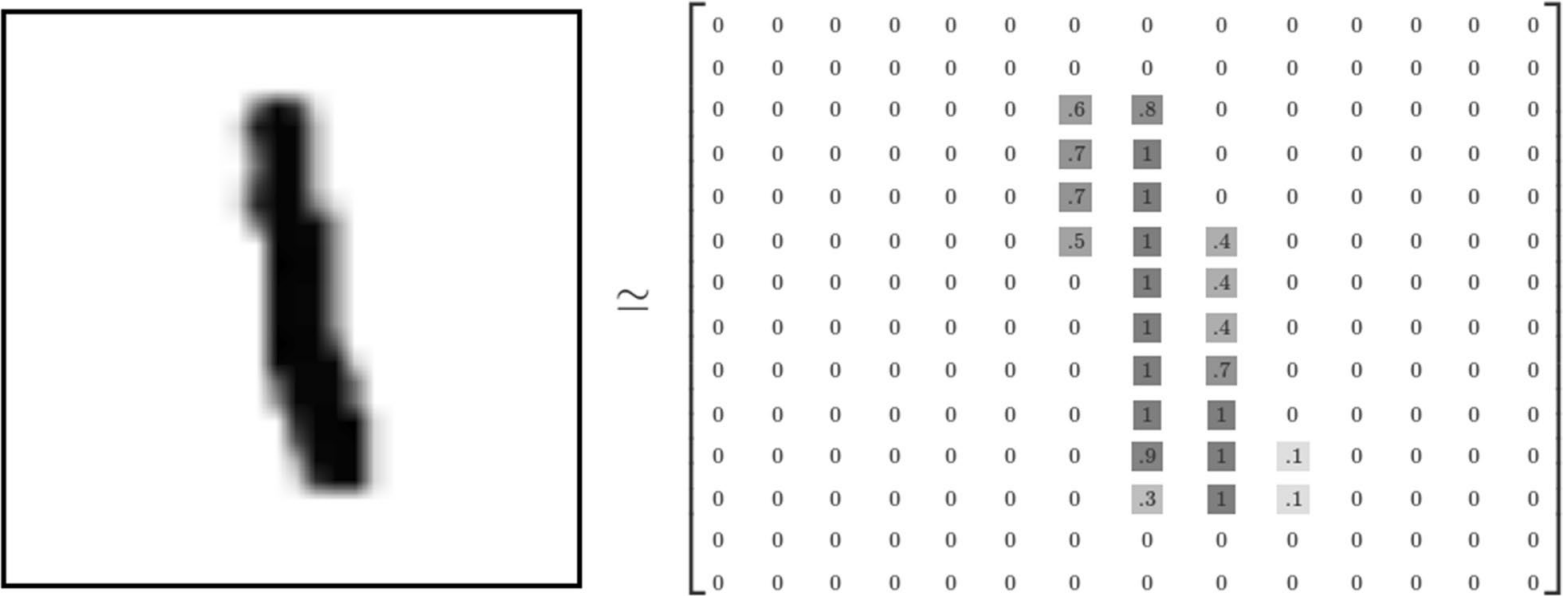
Matrix representation of a 8-bits black and white image. The values range from 0 to 255

**Fig. 3:**
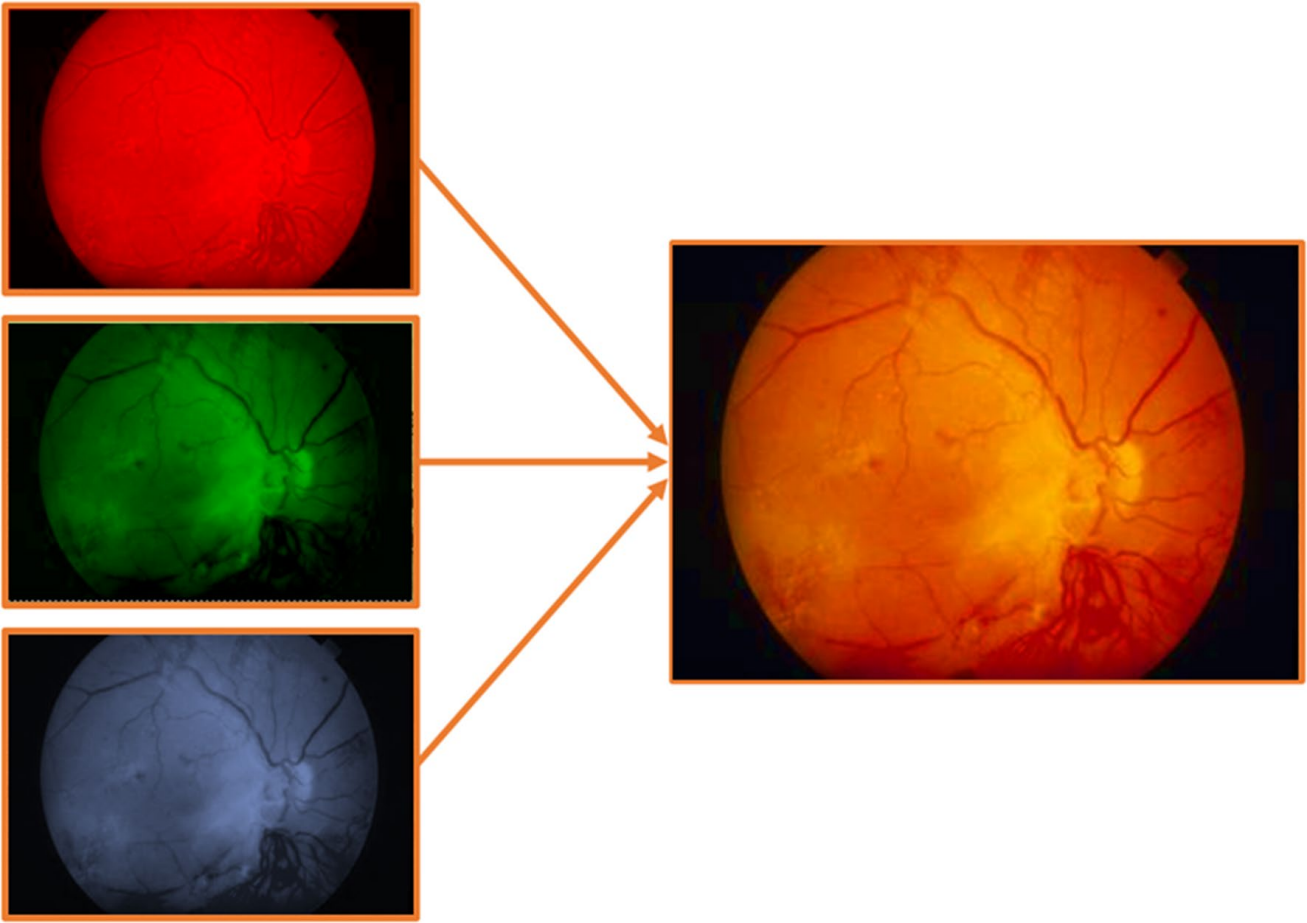
The three color channels (red, green, blue) of a retina image for diabetic retinopathy detection

In order to understand how machine learning algorithms and, more specifically Convolutional Neural networks, work with images, one needs to understand how computers actually *see* these images. Figure 2 shows the matrix representation of a black and white image [2]. Since this is a 8 bits image, the pixels can take values from 0 to 255 (2^8^ = 256) depending on the tonality of gray. For the images in color, the matrix representation would be the same, but instead of a single matrix, there will be three matrices corresponding to the red, green and blue color whose values will also range from 0 to 255. The mix of these three color channels will form the final image as can be seen in Fig. 3.

**Fig. 4:**
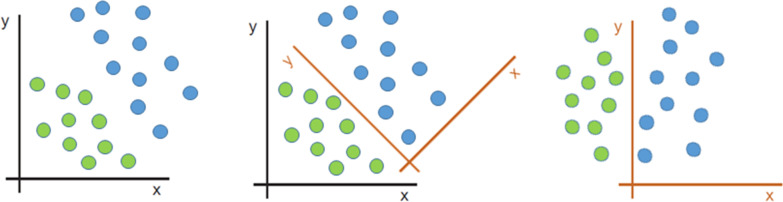
Solving a two-dimensional classification problem by eye by changing the data representation

Now that we know how computers represent images, let us do a small exercise. Let us take for instance the left image in Fig. 4. Imagine you need to separate the green points from the blue points. This would be what we have previously defined as a classification problem. Just by looking at the image any person would imagine the straight line separating both categories. But, what happens if you are told to give the equation of this straight line? Since there are no graduated axes and probably you have not done this exercise in quite a longtime, probably it will take you a couple of minutes to figure out which is the answer. Let us now imagine that we rotate and translate the axes as in the central image in Fig. 4. For more clarity, the right image in Fig. 4 shows the new x axis parallel to the text. The problem has not changed. Just the representation of the data has changed by performing a linear transformation. If we are asked now to separate green points and blue points, we would not even need 10 s: points with *x* > 0 are blue, and points with *x* < 0 are green. What has happened here? By changing the representation of the data, we have transformed a slightly difficult problem into a much easier problem to solve. This concept of representation change is paramount when understanding how Machine Learning and Deep Learning algorithms work. Since we have already seen that, for a computer, an image is just a set of values forming a matrix, we can easily understand that there is no much difference between the two-dimensional example in Fig. 4 and a multidimensional image matrix. Thus, our goal in an image-based classification problem would be to find alternative representations of the raw image values until we find a new way of looking at the data where the problem becomes easier. The two-dimensional problem in Fig. 4 was very easy to solve by eye, but in real live problems are much more complicated. This means that we would like to have some way for efficiently and systematically finding and testing new representations until we find the optimal one. This process of automatically searching for the optimal solution (or representation) for our problem is exactly what the Machine Learning algorithms do.

## Neural networks

**Fig. 5:**
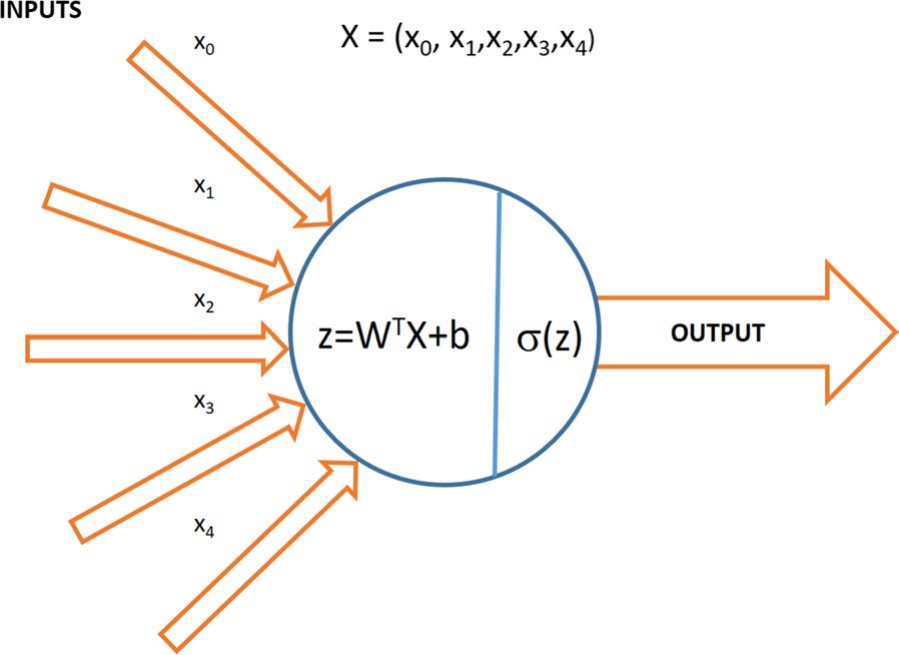
Structure of a single neuron in an artificial neural network

Artificial neural networks offer a way of achieving a systematic search for the optimal solution to the problem to be solved. Their elementary units, the neurons, are slightly inspired by the biological neurons: an artificial neuron receives one or more inputs (corresponding to postsynaptic potentials of the biological neurons dendrites in the biological analogy) and sums them to produce an output (or activation, representing a neuron’s action potential which is transmitted along its axon). Each input is separately weighted, and their sum is passed through a nonlinear function known as activation function. Without the activation function, the neural network could only solve linear problems. These functions work similarly to the threshold potentials needed to regulate and propagate signaling in the nervous system. The choice of these activation functions is one of the *hyperparameters* in a learning algorithm, which means that it is up to us to decide which one to use. Figure 5 shows the structure of an artificial neuron.

**Fig. 6:**
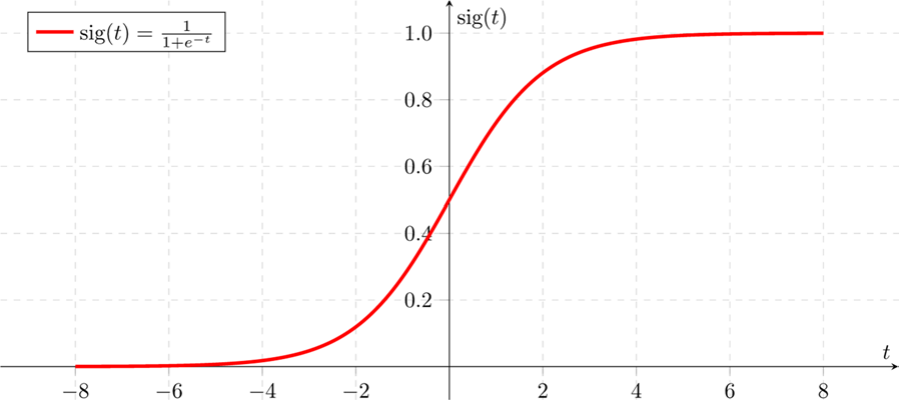
Sigmoid function

The* W* matrix and the* b* or bias factor are first applied to the input data *x* in order to perform a linear transformation (rotation + translation, respectively) similarly as in the 2D example in Sect. 3. The *σ* in this case is a particular and widely used activation function called sigmoid. The shape of the sigmoid function can be seen in Fig. 6. The sigmoid gives smoother output values than a simple step function, is differentiable and presents a very nice property: its derivative depends on the function itself.

$$\sigma^{^{\prime}} (x) = \sigma (x) * ({1} - \sigma (x))$$

We will later see that this is a desirable feature for an activation function.

### Learning process

**Fig. 7:**
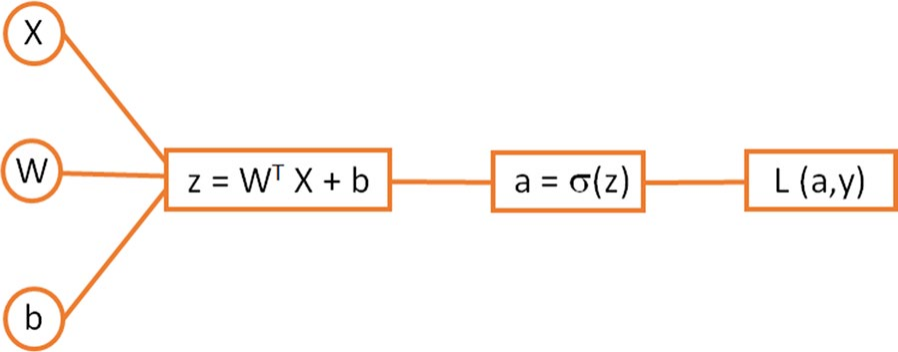
Forward propagation of a single neuron

Let us now see how the learning process works. For most of its range, the sigmoid function will return values close to 1 or close to 0. This makes this function very appropriate for binary problems, like in the case of the classification problem in Fig. 4. The output of the activation function can then be taken as the answer to the problem: an output value of 0 can correspond to blue and a value of 1 to green, or the other way around: it does not matter as long as we keep the criteria consistent. We can take each of the data points in Fig. 4 and pass it through the neuron. In this case, the input is bidimensional corresponding to the x and y coordinates of each of the data points, i.e., the input value is *X* = (*x*_input_, *y*_input_). Taking one set of values from our input dataset through one single neuron (also called forward propagation), the full expression is shown in Fig. 7.

where the *W* matrix and the bias *b* correspond, in this first iteration, to some random values. The *L* is the so-called loss function. This is a function accounting for how well we are performing. It depends on *a* (the prediction) and *y* (the true label of the data points, i.e.: 0 in the case of blue and 1 in the case of green). This is where the labels in the supervised learning approach come into play. In order to learn with a supervised algorithm, we need to know beforehand the category of our data points. The* L* should be small when we are performing well (i.e., predicting properly the category of each data point) and should be big when we are not doing a good job. This means that, for an optimal solution, the loss function should be as small as possible. This transforms our learning problem into a minimization problem. We need to minimize the loss function.

This minimization process is what we call *learning*. One can decide which is the loss function depending on the problem to be solved, for instance, as an example, the loss function can just be the mean squared error between the prediction *a* and the real label *y*. The mean squared error is calculated as the average of the squared differences between the predicted and actual values or labels, i.e., Σ(*a*_*i*_ − *y*_*i*_)^2^. The result is always positive, and a perfect algorithm will give a value of 0. The squaring means that larger mistakes result in more error than smaller mistakes, i.e., the model is punished for making larger mistakes. Another possible choice for loss function is the cross-entropy function, widely used for classification problems. For the educational purpose of this document, understanding the mean squared error would be enough.

**Fig. 8:**
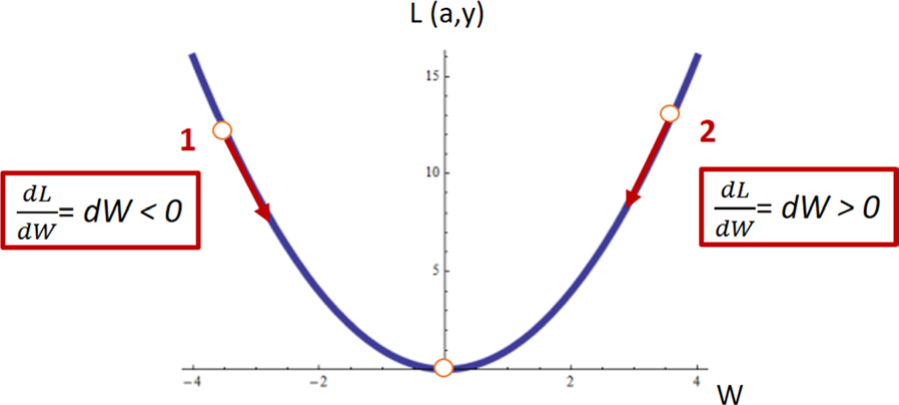
Minimization of a parable. The random *W* value at point 1 presents a negative slope while at point 2 presents a positive slope

After the first pass through the neuron with random values for *W* and *b*, the second iteration will not be random at all. We want to learn which are the values of *W* and *b* so that *L* is as small as possible. For this, we will calculate the derivatives and update the values of *W* and *b* according to the direction of the minimum. This can be better understood taking a look at Fig. 8 where a hypothetical curve of *L* versus *W* is plotted. After the first iteration of our neuron, the *W* value can be either greater or smaller than the *W* minimizing the *L* function (*W*_min_). For this particular problem, if the *W* value is smaller than *W*_min_ (point 1 in Fig. 8), the slope at that point, and hence, the derivative d*L*/d*W*, would be negative. In the same way, if the *W* value is greater than *W*_min_ (point 1 in Fig. 8), the derivative at that point would be positive. This means that, in both cases, if we subtract the derivative from the initial random value of* W*, we would be going in the direction of the *W*_min_ as indicated by the red arrows in Fig. 8.

**Fig. 9:**
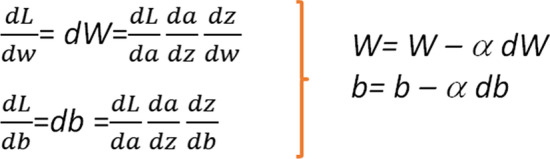
Derivative calculation using the chain rule and update of the* W* and* b* parameters in the direction of the minimum

The same is valid also for the value of *b*. After the first iteration, the values of *W* and *b* can be updated by the formula in Fig. 9. The computation of the gradient of the loss function with respect to the weights of the network (*W* and *b*) is called backpropagation. The term *α* in Fig. 9 is known as the learning rate. The derivative gives the direction of the step in the direction of the minimum, and the learning rate gives the magnitude of the step. *α* is another of the model *hyperparameters.* In the case of *α*, we must take into account that if it is too large, we may take steps that are too big and we may miss the minimum. If the learning rate is too small, the learning process will be too slow since we will be approaching the minimum with tiny steps.

The process will be then repeated with all the points in Fig. 4, updating for each iteration the values of *W* and *b* so that they come always closer to the ones that minimize the loss function, optimizing thus the performance of the algorithm.

### Multilayer neural networks

**Fig. 10:**
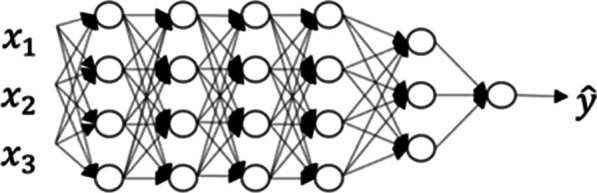
Neural network with 5 hidden layers

For very simple problems, one single neuron can be enough. Usually in a real-life problem, one needs more than one data representation change in order to solve it. This is where the term *network* comes into play. For complicated problems, one would need many different representations of the data, that will be combined among them to create further representations, that will in turn combine, etc., in order to reach the optimal representation allowing to solve the image classification problem or any other problem one may want Machine Learning to solve. This stack of combined representations can be nicely visualized with the shape of a network. Figure 10 shows one of these neural networks, where each of the connected nodes represents one neuron as the one showed in “Convolutional Neural Networks” section. We call this type of networks fully connected neural networks since all the neurons in one layer are connected with all the neurons in the following layer. The *layer* of a neural network is a collection of neurons operating together at a specific depth within a neural network. The input layer contains the raw data. The hidden layers are the ones between input layers and output layers. The number of hidden layers is another model hyperparameter to be chosen during optimization. Figure 10 represents a neural network with 5 hidden layers.

The optimization process described in the previous section, where a function is minimized by iteratively moving in the direction of steepest descent as defined by the negative of the gradient, is called gradient descent.

The learning process described for a single neuron would be the same in a neural network with several layers. The expressions are similar to the ones in Fig. 9, the fact of having many layers will be reflected in the amount of terms in the different expressions when applying the chain rule to calculate the gradient, but the procedure will be similar as in the case of just one neuron.

We have based our example in a 2D problem for clarity purposes, but everything explained here can be trivially generalized to a *N*-dimensional dataset.

### Some additional comments

It can be observed that all the expressions in this section are written in matrix form. This is one of the advantages of the gradient descent method: it allows to compress the equations with a very simple notation. Besides, many programming languages work optimally with matrices, speeding dramatically the calculations with respect to looping over all the variables at every iteration. Also, as it was previously mentioned, choosing activation functions whose derivative depends on the function itself allows to have the value of the derivative calculated when performing the backpropagation (explained in “Learning process” section), since it was already obtained in the forward propagation step. This greatly improves the computational performance of the learning algorithm.

On the other hand, an appropriate choice of the model hyperparameters plays a key role in the success of a neural network model. The learning rate, the type of activation functions and the number of hidden layers have already been mentioned as some of the main ones. Another hyperparameter that is worth mentioning is the number of epochs which indicates the number of times that the learning algorithm will work through the entire training dataset. Datasets are usually grouped into batches, especially when the amount of data is very large. During the learning process, the value of the model weights is updated every time the algorithm works through each of the data batches. The amount of data from the original dataset in each of the batches is also a hyperparameter to be set by the machine learning practitioner.

### Why the *deep* in deep learning?

**Fig. 11:**
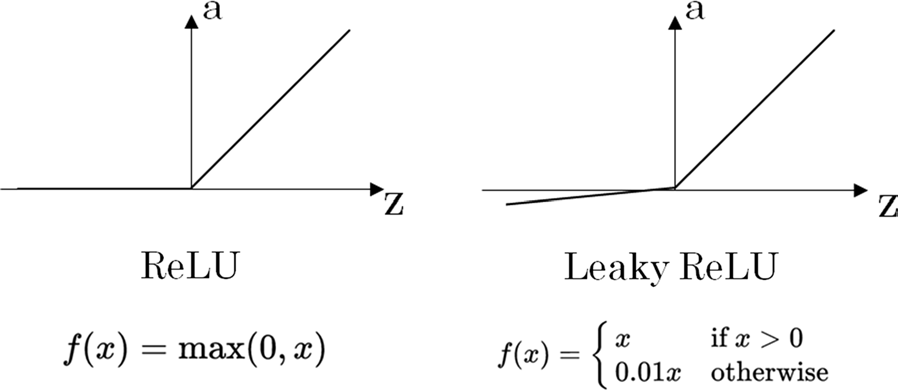
ReLU and leaky ReLU activation functions
- Convergence problems: a fast convergence of the gradient descent algorithm to the minimum is not guaranteed. The optimization algorithm to be used is another hyperparameter and a correct choice can mean the difference between good results in minutes, hours, or days. Several optimization algorithms have been developed to improve the convergence problems, such as the Adam optimization [4]. This algorithm uses the squared gradients to scale the learning rate and it takes advantage of adaptive momentum by using the moving average of the gradient instead of gradient itself.

The depth of a neural network is its number of neuron layers. Deep Learning refers to the fact of having many more layers than in the so-called Machine Learning algorithms together with all the issues arose from it. The philosophy is the same for both cases but, having more layers usually implies further problems. We summarize here three of the most common issues in Deep Learning:

- Vanishing gradient: the sigmoid activation function presents derivatives very close to zero for most of its range. This is not important when having a few layers, but for very deep neural networks, the product of many values too close to zero can result in a neural network that is unable to learn. This is easy to understand taking a look at the expressions in Fig. 9 and imagining what happens if the *da/dz* factor is very small. For Deep Learning algorithms, specific activation functions are used, such as ReLU [3] or leaky ReLU (see Fig. 11), where the derivatives values (and hence the gradients) are greater than for a sigmoid. Each problem is different, but a rule of thumb can be to use ReLU (or leaky ReLU) for the hidden layers and use the sigmoid only for the last layer when working on a binary classification problem.
- Overfitting problems: Overfitting occurs when a good fit is achieved on the training data, while the model does not generalize well on new, unseen data. This means that the model has learned patterns specific to the training data, which are irrelevant to other new or different data. This can also happen in Machine Learning algorithms, but are more of an issue for Deep Learning models due to the larger amount of neurons. It can be easily understood thinking about the number of neurons as if it were the degree of a polynomial: the greater the degree, the easier it is to find a curve passing by all the N points. But if we add a new point to the dataset, most probably the perfect N-points fit would fail on the *N* + 1 point, meaning that the model is not general enough for this specific problem. Several regularization methods have been developed to avoid the overfitting, such as dropout, data augmentation or *L1*/*L2* regularization. The dropout technique consists of randomly dropping out nodes during the training to avoid the over specialization of some neurons. The data augmentation technique is used to increase the amount of data by adding slightly modified copies of already existing data or newly created synthetic data from existing data, so that the algorithm does not *see* the same data twice. When working with images, the modifications can be geometric transformations, flipping, color modification, cropping, rotation, noise injection, random erasing, etc.
  The *L*1/*L*2 regularization techniques consist of adding a penalty term to the loss function: the absolute value of the magnitude of the network weights for L1 and the squared magnitude for *L*2.
  When minimizing this modified loss function, the penalty term (that is always positive since it is either a magnitude or its squared value) will advantage network weights as small as possible while still trying to optimize the network performance. This will help the neural network to regularize itself, since it will favor simpler models. This can be understood again in terms of a fit to a polynomial: trying to have smaller polynomial coefficients will lead to a simpler model that will not try to perfectly fit every single outlier in the training dataset, but to give a more general result that will potentially have a better performance when exposed to new data.
  In any case, the generalization to new data is still one of the major problems of AI, especially in the medical context where datasets are sometimes not as large, varied and balanced as desirable.

## Convolutional neural networks

Until now, we have described how the learning algorithms work on general *N*-dimensional data points, but all what has been explained previously can be applied to images. The computational structure of images was described in Sect. 3, and it was already explained that, at the end of the day, images are just matrices and can be treated in the same way as we have shown in the previous sections. Nevertheless, to deal with images we must introduce Convolutional Neural Networks. This type of Neural Networks has established the state of the art in computer vision since 2012, when they beat all their competitors at the ImageNet Large Scale Visual Recognition Challenge in 2012 [5].

**Fig. 12:**
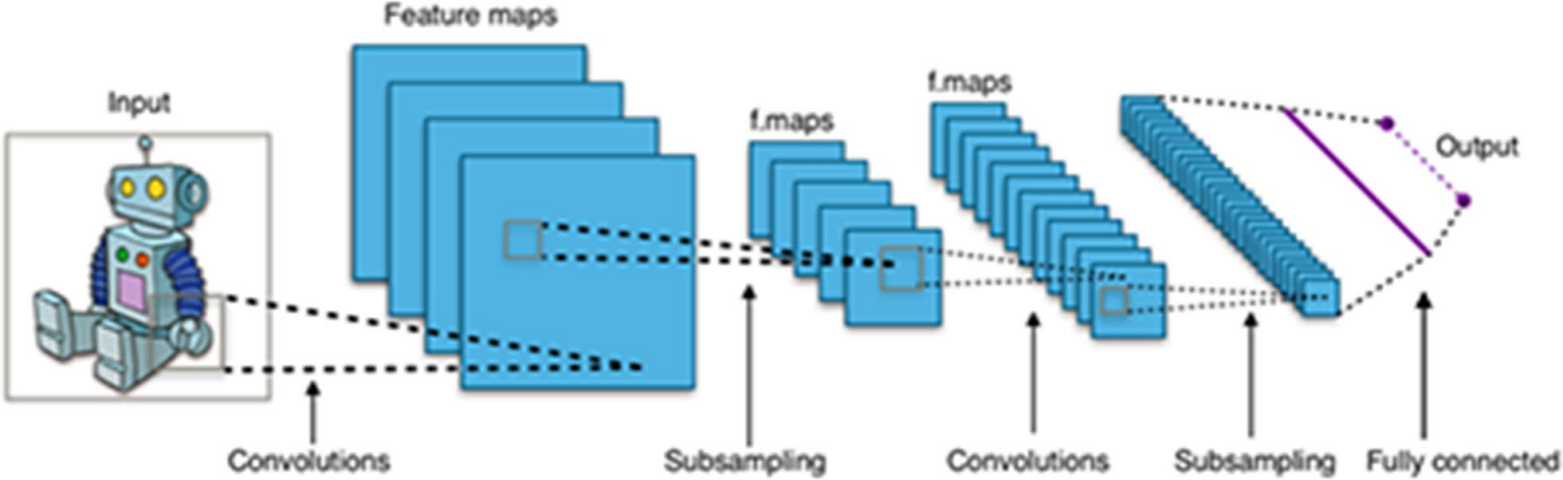
General convolutional neural network architecture

The convolutional neural networks employ a specialized kind of linear operation called convolution. Convolutional networks are simply neural networks that use convolution in place of general matrix multiplication in at least one of their layers. Figure 12 shows the general architecture of a ConvNet where multiple filters are taken to slice through the image and map them one by one learning different portions of an input image. One can imagine a small filter sliding left to right across the image from top to bottom and that moving filter is looking for, say, some vertical edge. Each time a match is found, it is mapped out onto an output image called feature map.

**Fig. 13:**
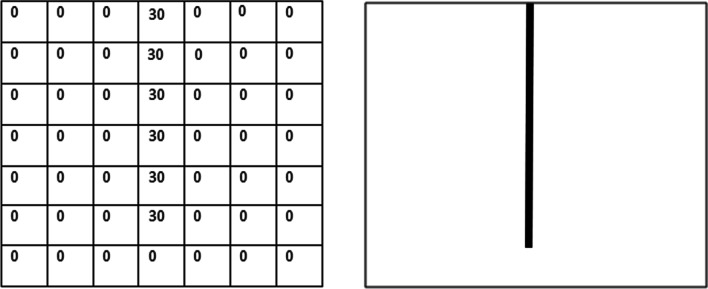
Pixel representation of a vertical line filter (left) and its visualization (right)

An example of a filter can be seen in Fig. 13. The image on the left shows the pixel representation of a vertical line filter and the image on the right shows its visualization.

**Fig. 14:**
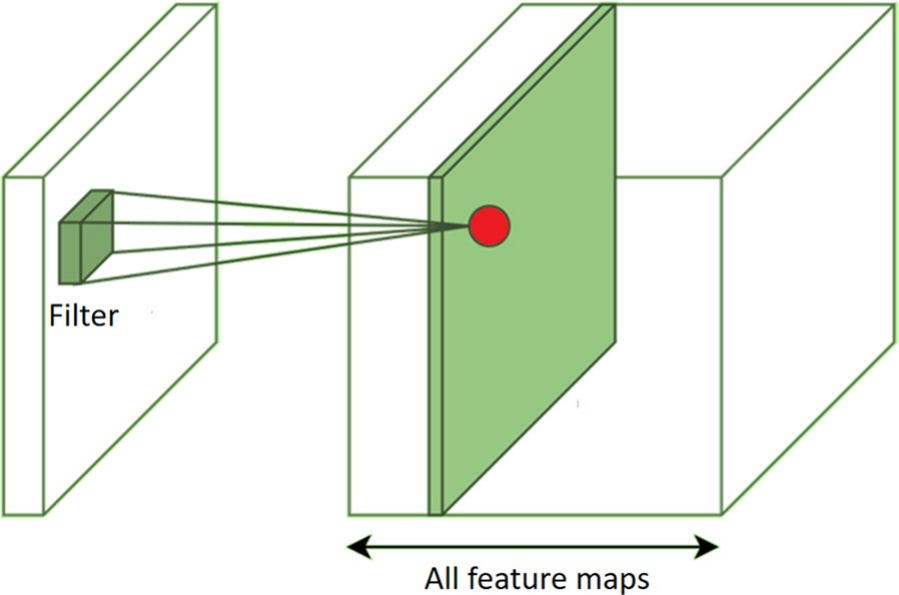
Extraction of features from the original image by using sliding filters

During the sliding through the input images in the neural network, the filter pixel values are multiplied by the pixel values of the image section. If the image presents some feature similar to the vertical line at that particular position, the result of the multiplication will be a high value scalar while, if the shape at that point is completely different from a vertical line, the resulting scalar will be of smaller value. The feature map is then a matrix formed by all these scalars, giving information on the presence and on the location of the particular feature: if the original input image had a vertical straight line on the top left corner, the corresponding feature map matrix will present greater values on that same corner. This means that, each feature map is the mapping of a certain feature in the original image (see Fig. 14). Hence, the convolutional part of a neural network creates a new representation of the original image by extracting and separating the main relevant features of it. By *main relevant features*, we mean here the optimal features for solving a certain problem such as how to distinguish the two different varieties of glioblastoma mentioned in Sect. 2 or the classification of the objects that a self-driven car has in front of it.

Since usually various dozens of filters are needed to solve an image classification problem and each filter generates a corresponding feature map that is given as input to the following layer in the neural network, the problem’s size can scale very quickly.

To alleviate this effect, there are several methods to reduce the dimensionality of the feature maps. One of the most widely used is the so-called *max-pooling*.

**Fig. 15:**
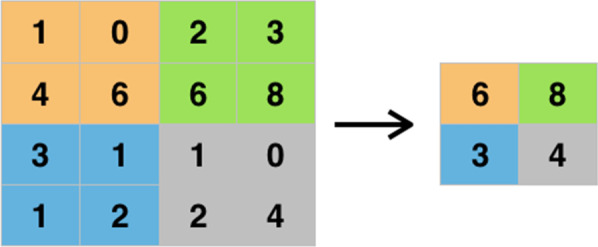
Max-pooling applied to a feature map, reducing its dimensionality

It consists on going through the feature map, usually with a sliding window of 2 × 2, taking the maximal pixel value on that square. A graphical illustration of this can be seen in Fig. 15.

Another well-known type of pooling is the *average pooling* which, instead of taking the maximal value, takes the average of all the values in the feature map.

For the convolutional neural networks, the filter values are the ones to be learnt (the *W* and *b* following our terminology). As for the fully connected neural networks, the first iteration will have random filters, and all the optimization process will take place by applying the gradient descent method until it reaches the optimal set of filters.

After the convolutional part where the features are extracted, these features are given as input to a fully connected network that will perform the classification as explained in previous sections. The fact that the features of interest are learnt during the optimization represents another great step forward with respect to more classical algorithms in computer vision, where some expert should first extract by hand the features under consideration for solving the problem (feature engineering) and then give them as input to the classification algorithm.

An interesting fact that makes the convolutional neural networks so competitive when dealing with images is that they are translation invariant. This means that if one shifts an image a bit, it would have a similar activation map as the image before shifting. This is because the convolution is a feature detector, independently of the position of the feature. The translation invariance of the convolutional neural networks allows to learn using only very few parameters with respect to classical methods.

### Transfer learning

The traditional supervised learning approach breaks down when we do not have sufficient labelled data for the task we care about to train a reliable model. This is often the case when dealing with medical images. Transfer learning is the capacity of learning a new task through the transfer of knowledge from a related task that has already been learned. This can be achieved thanks to the hierarchical way in which the neuronal networks learn. The first layers in a model working to classify images will learn very general visual features such as intersections, straight lines, simple curves, dots. These basic image components are common to any type of picture we will be working with. As one goes deeper into the layers of the model, the algorithm will extract more complex features that are also more specific of the particular problem being studied.

This means that we can train a model to perform image classification using a big dataset (typically ImageNet [6]) and then re-use it to perform another task where we do not have that many data. For doing so, the first layers of the neural network can be left frozen during the new training, i.e., the filter weights will not change. The small dataset will then be only used to learn the filter weights on the last layers who are in charge of the problem specific features extraction. This allows to make a better exploitation of small datasets, fully using the relevant information to extract the most meaningful features and *transferring* the most general parts of the model from a different problem where more data are available. This technique is widely used nowadays in the medical field. As an example, an extensive review of its use for the diabetic retinopathy case can be found at [7].

## Limitations

There is no doubt that deep learning has managed to achieve a very important scientific milestone in many different areas, including medicine. But these new techniques still present some limitations to be widely used in the clinical practice. One of the problems come from the variability of the data itself (e.g., contrast, resolution, signal to noise) which make the Deep Learning models suffer from a poor generalization when the training data come from different machines (different vendor, model, etc.) with different acquisition parametrization or any underlying component that can cause the data distribution to shift. These over-parametrized models have a high tendency to rely on superfluous correlations and are very sensitive to any shift caused by external factors such as different scanner or acquisition protocols.

This generalization gap can be partially mitigated through some techniques. Some of them have already been introduced in this article, and some of them are still being widely explored. The easiest one relies on the fact that all deep learning methods perform better when there is more data to train the model. This is also a problem in medicine, since it is not easy for single sites to generate a large amount of data and manual labels. Working in multi-center initiatives and crowd sourcing can be an efficient approach to achieve such useful resources. Other approaches that will have a great impact in this kind of problem rely on generative models. One of the disadvantages is that the most prominent generative models such as generative adversarial networks (GANs) [8] usually require a large amount of data, and it can take non-desirable shortcuts to model the underlying distribution. Recent likelihood-based models [9] showed some improvements; however, it is still very difficult to model such high dimensional distributions. Another approach, also concerning unlabelled data, is the use of semi-supervised learning methods, that can yield improvements even when working with small datasets. A possible way to minimize the problem of creating a great amount of manual annotations is to use active learning, where the most uncertain predictions are selected for manual correction before re-training the model.

Another big issue is the lack of model interpretability and explainability of the deep learning models. This is common to all areas, but in some of them, such as medicine and health care, not addressing such challenge might seriously limit the chances of adoption, in real practice, of computer-based systems that rely on these complex nonlinear models. Currently, many techniques tackling this very important issue are being explored and great advances are being achieved in this direction [10].

Finally, it is necessary to address one of the main problems that is transversal to all the other mentioned issues: the feedback loop between the deep learning practitioner and the health professional is paramount to be able to make real advances and to build robust models both from a mathematical and from a medical point of view. Projects with a multidisciplinary approach, containing people from different domains, are thus essential to make the most of these very powerful techniques and to be able to use them confidently in a clinical routine.

## Conclusions

This document summarizes for non-deep learning experts and clinicians in particular, the main aspects to understand how neural networks work, placing emphasis on the convolutional neural networks that represent the state-of-the-art algorithms for image analysis. The concepts of automatic learning and data representation have been reviewed together with the functioning of the neurons in a neural network and the main advantages and problems, emphasizing that deep learning is not just a multilayered machine learning approach, but it also has to do with the consequent improvement and optimization of the learning algorithms to make neural networks more robust. The intention of this work was to introduce the basics and set a strong foundation for clinicians on the topic so that they can continue building on top of it with more advanced concepts.

## Data Availability

Not applicable.

## Abbreviations

AI: Artificial intelligence
ConvNet: Convolutional neural network
MRI: Magnetic resonance images
